# Metagenomics-based exploration of key soil microorganisms contributing to continuously planted *Casuarina equisetifolia* growth inhibition and their interactions with soil nutrient transformation

**DOI:** 10.3389/fpls.2023.1324184

**Published:** 2023-12-06

**Authors:** Yuhua Wang, Shaoxiong Lin, Jianjuan Li, Xiaoli Jia, Mingyue Hu, Yuhong Cai, Pengyuan Cheng, Mingzhe Li, Yiling Chen, Wenxiong Lin, Haibin Wang, Zeyan Wu

**Affiliations:** ^1^ College of JunCao Science and Ecology, Fujian Agriculture and Forestry University, Fuzhou, China; ^2^ Fujian Provincial Key Laboratory of Agroecological Processing and Safety Monitoring, College of Life Sciences, Fujian Agriculture and Forestry University, Fuzhou, China; ^3^ College of Life Science, Longyan University, Longyan, China; ^4^ Editorial Department, Fujian Academy of Forestry Survey and Planning, Fuzhou, China; ^5^ College of Tea and Food, Wuyi University, Wuyishan, China

**Keywords:** *C. equisetifolia*, continuous planting, metagenome, nutrient transformation, soil enzymes, gene function

## Abstract

*Casuarina equisetifolia* (*C. equisetifolia*) is an economically important forest tree species, often cultivated in continuous monoculture as a coastal protection forest. Continuous planting has gradually affected growth and severely restricted the sustainable development of the *C. equisetifolia* industry. In this study, we analyzed the effects of continuous planting on *C. equisetifolia* growth and explored the rhizosphere soil microecological mechanism from a metagenomic perspective. The results showed that continuous planting resulted in dwarfing, shorter root length, and reduced *C. equisetifolia* seedling root system. Metagenomics analysis showed that 10 key characteristic microorganisms, mainly *Actinoallomurus*, *Actinomadura*, and *Mycobacterium*, were responsible for continuously planted *C. equisetifolia* trees. Quantitative analysis showed that the number of microorganisms in these three genera decreased significantly with the increase of continuous planting. Gene function analysis showed that continuous planting led to the weakening of the environmental information processing-signal transduction ability of soil characteristic microorganisms, and the decrease of *C. equisetifolia* trees against stress. Reduced capacity for metabolism, genetic information processing-replication and repair resulted in reduced microbial propagation and reduced microbial quantity in the rhizosphere soil of *C. equisetifolia* trees. Secondly, amino acid metabolism, carbohydrate metabolism, glycan biosynthesis and metabolism, lipid metabolism, metabolism of cofactors and vitamins were all significantly reduced, resulting in a decrease in the ability of the soil to synthesize and metabolize carbon and nitrogen. These reduced capacities further led to reduced soil microbial quantity, microbial carbon and nitrogen, microbial respiration intensity, reduced soil enzyme nutrient cycling and resistance-related enzyme activities, a significant reduction in available nutrient content of rhizosphere soils, a reduction in the ion exchange capacity, and an impediment to *C. equisetifolia* growth. This study provides an important basis for the management of continuously planted *C. equisetifolia* plantations.

## 1 Introduction

Continuous planting refers to continuous monoculture of the same plant on the same land, which is common in agricultural production due to limited land resources (Jing et al., 2022; Sumberg and Giller, 2022). However, continuous planting is highly susceptible to changes in the soil environment, which in turn affects plant growth and is not conducive to sustainable agricultural development (Hufnagel et al., 2020; Tan et al., 2021). *C. equisetifolia* is an economically important tree species, which is mainly distributed in coastal areas of Guangdong, Fujian, and Hainan provinces in China (Zhong et al., 2010). *C. equisetifolia* with a well-developed root system is highly resistant and can reach a height of up to 30 m and a diameter of up to 70 cm at breast height, and can be harvested within 10 years of planting; in addition to being an economically important forest tree, *C. equisetifolia* is also commonly used in coastal areas for soil and water conservation, protection against tidal erosion, etc. (Athikalam and Karur Vaideeswaran, 2022). The ecological environment of coastal sandy areas is more specialized, and there are fewer tree species that have some economic value and are suitable for planting in sandy environments; therefore, *C. equisetifolia*, as a major planting species, has been planted for a long period in coastal areas, and it can only continue to be replanted in-situ, even after it is harvested (Anbarashan et al., 2022). As the number of continuous monoculture of *C. equisetifolia* continues to increase, the phenomenon of continuous planting becomes more and more pronounced, as evidenced by a decrease in tree height by 23.7%, diameter at breast height by 24.4%, and volume by 29.0% (Zhang et al., 2000). Continuous planting has already had an impact on growth, severely limiting the sustainability of *C. equisetifolia*’s protected forest resources.

Soil is a carrier for plant growth, and microorganisms are extremely important in plant-soil interactions for soil nutrient effectiveness and plant access to nutrients (Zhao M. et al., 2021; Ye et al., 2023b). In agricultural production practice, long-term continuous monoculture causes serious harm to soil physicochemical and microbial communities, as evidenced by a decrease in soil bacterial diversity and richness indexes with an increase in the number of continuous planting years, and a decrease in soil fertility (Chen et al., 2022a; Guo et al., 2022b). The reason for this change is that soil microorganisms are driven by continuous planting and the microbial community evolves in a single direction, leading to simplification of microbial community diversity and function, deterioration of soil physicochemical characteristics, exacerbation of pests and diseases, and impediment to plant growth (Chen et al., 2022b). Second, changes in soil microorganisms after long-term continuous planting of plants directly affect extracellular enzyme activities in the soil, which in turn alters nutrient cycling and available nutrient content in the soil and reduces nutrient uptake by plants (Wang et al., 2022b). It has been reported that continuous planting of tea trees is associated with a massive increase in pathogenic bacteria in the soil, a decrease in the number of probiotic bacteria, a decrease in enzyme activity associated with soil nutrient cycling, a decrease in the content of available nutrients, and a significant reduction in the yield and quality of tea leaves (Lin et al., 2022). Long-term continuous planting of alfalfa results in significant changes in the abundance of Actinobacteria, Gammaproteobacteria, Armatimonadetes, Chloroflexi, Firmicutes, and Gemmatimonadetes in rhizosphere soil, a significant reduction in soil available phosphorus, and a significant decrease in alfalfa yield (Xu et al., 2022). Continuous planting of ginseng tends to increase the abundance of *Fusarium* spp. and reduce the abundance of *Burkholderia* spp. in soils, which significantly reduces the carbon cycling capacity of the soil, decreases soil fertility, and increases the incidence of root disease, leading to a significant reduction in ginseng yield and quality (Liu et al., 2022). It can be seen that the key reason for the effect of continuous planting on plant growth is caused by changes in soil texture due to changes in soil microbial ecosystems (Pang et al., 2021b; Wu et al., 2021).

Currently, studies on continuously planting obstacles of artificial timber forests have mainly involved *Cunninghamia lanceolata, Eucalyptus robusta, Larix gmelinii*, and *Picea koraiensis*, but there are still fewer studies on *C. equisetifolia*, and the causes of continuously planting in *C. equisetifolia* plantations are still being explored, especially the effects of continuously planting on soil microorganism communities and functions (Liu et al., 2020; Li et al., 2021b; Xu et al., 2021; Ma et al., 2023). Our research team found that after continuous planting, the expression capacity of nitrogen transformation genes in *C. equisetifolia* rhizosphere soil was significantly reduced, available nitrogen content in the soil decreased, and *C. equisetifolia* growth was inhibited, and suggesting that this phenomenon may be closely related to changes in soil microbial diversity and function (Zhou et al., 2021; Zhou et al., 2022). It is well known that the plant root system, when sensing changes in the external environment, affects the number, species and function of rhizosphere microorganisms through the release of root secretions, which in turn alters the nutrient cycling of rhizosphere soil to adapt to the environment (Shen and Lin, 2021; Tan et al., 2022). Therefore, in-depth analysis of the effects of continuous planting on the diversity of rhizosphere microbial communities and their functions is of great significance in revealing how continuous planting affects rhizosphere microbes, which in turn alters soil nutrient cycling and inhibits *C. equisetifolia* growth.

Soil metagenomics technology, which can be used to analyze the complete full microbial genetic composition of soils and their community functions, has been widely used (Semenov, 2021). In recent years, many scholars have made significant progress in analyzing the effects of continuous planting on the diversity of soil microorganisms and their functions in the rhizosphere zone of different plants by using soil metagenomics technology, which has made important contributions to the reduction of continuous planting obstacles (Pang et al., 2021a; Gu et al., 2022; Li et al., 2023). However, the use of soil metagenomics to analyze the effects of soil organisms on soil extracellular enzyme activities and nutrient cycling in the *C. equisetifolia* root system has rarely been reported, and an in-depth understanding of this mechanism is of great significance for the cultivation and management of *C. equisetifolia* plantations. Accordingly, this study was conducted to collect root soil and rhizosphere soil of *C. equisetifolia* tree with different numbers of continuous plantings to analyze the effects of continuous plantings on *C. equisetifolia* seedling growth and nutrient transformations in rhizosphere soil. At the same time, soil enzyme activities, soil microbial biomass carbon, nitrogen and respiration intensity, and soil bacterial, fungal and actinomycete quantities were measured to analyze the effects of continuous planting on nutrient transformation and microbial community in *C. equisetifolia* tree rhizosphere soil. In addition, soil metagenomic techniques were used to analyze soil microbial community diversity and its functional changes, and to analyze the effects of continuous planting on soil microbial community structure and their functions in the *C. equisetifolia* tree rhizosphere zone. On this basis, the interactions between soil nutrient transformations and soil enzymes, soil microbial communities and functions were further analyzed, with a view to revealing the soil ecological mechanisms that impeded *C. equisetifolia* trees growth, and to providing some references for the cultivation and management of continuously planted *C. equisetifolia*.

## 2 Materials and methods

### 2.1 Experimental design and sample collection

The experimental site where this study carried out was located in a national shelterbelt forest in Chihu Township, Hui’an County, Fujian Province, China (118°55′ E, 24°35′ N). The average annual temperature of this forest is 19.8°C, the annual rainfall is 1,029 mm, the soil texture is wind-deposited yellow sand, the total area is about 433 ha, and the main plantation is *C. equisetifolia* tree with a planting density of 950 plants/ha. *C. equisetifolia* seedling was first planted in this forest in 1987, and replanting continued *in situ* after the forest cut down some *C. equisetifolia* trees in 2011. In March 2018, three different plots were selected at the forest site that had not been planted with *C. equisetifolia*, had been planted with *C. equisetifolia* once, and had been planted with *C. equisetifolia* twice. After the above three plots were replanted with *C. equisetifolia* seedlings (height 0.8 m, planting density 950 plants/ha), three different numbers of continuous plantings of *C. equisetifolia* seedlings were formed in this study, namely the first planting of *C. equisetifolia* (M1), the second continuous planting of *C. equisetifolia* (M2) and the third continuous planting of *C. equisetifolia* (M3). Each plot had a total area of 2700 m^2^ and was set up in three separate areas of 900 m^2^ (30 m × 30 m) each, i.e. three independent replicates. The management of *C. equisetifolia* seedling during planting was harmonized in accordance with the normal management techniques from 1987 to 2018, and with the “Technical Regulation on the cultivation of casuarina seedlings and trees” (LY/T 3092-2019) since 2019 (National Forestry and Grassland Administration of the People's Republic of China, 2019).

In March 2022, root soil and rhizosphere soil were collected from different numbers of continuous plantings of *C. equisetifolia* trees (M1, M2, and M3), and rhizosphere soil was used to analyze soil cation exchange, effective nutrient content, soil enzyme activity, soil microbial biomass carbon, nitrogen, and respiration intensity, soil bacterial, fungal, and actinomycete counts, and soil metagenomics, whereas root soil was used to potting *C. equisetifolia* seedlings to analyze the effect of soil on the growth. Rhizosphere soil was sampled using the “S” sampling method (Wang et al., 2020), i.e., 20 plants of *C. equisetifolia* trees were randomly selected for each replicate, the dead branches, leaves litter and other residues were removed, the upper layer of soil was shoveled out layer by layer for about 30 cm, the fine roots were cut and gently shook, and the soil that was still adhering to the roots was collected and mixed, i.e., it was the rhizosphere soil, and one replicate totaled about 500 g of rhizosphere soil. Three independent replicates were established for each treatment. The collected rhizosphere soil was placed on dry ice and brought back to the laboratory and immediately tested in the experiment. Root soil was sampled by randomly selecting 20 plants of *C. equisetifolia* trees, removing the deciduous layer, shoveling the upper layer of soil about 10 cm layer by layer, and collecting about 60 kg of soil centered on the main trunk of *C. equisetifolia* trees, with a radius of 10 cm and a depth of 10-40 cm, i.e. one replicate, and setting up three independent replicates for each experimental treatment.

### 2.2 *C. equisetifolia* seedling pot experiment and growth trait measurement

A pot experiment was used to analyze the effect of continuously planted soil on the growth of *C. equisetifolia* seedlings. The *C. equisetifolia* tree root soil collected from each treatment was thoroughly mixed, and removed residues, and packed into 24 cm diameter, 25 cm high pots with 9 kg of soil per pot. *C. equisetifolia* seedlings (28 cm in height and 2 years old) with relatively uniform growth were transplanted into pots of 2 plants each. Three treatments (M1, M2, and M3) with three replicates each and four pots per replicate, totaled 36 pots and 72 plants. Potted *C. equisetifolia* seedlings were placed outdoors in the glasshouse (mean temperature 25 °C, humidity 85% and light intensity 5000 Lux) and watered once a day in the evening with 200 mL of water per pot per time during planting. In May and November 2022, *C. equisetifolia* was fertilized uniformly with a compound fertilizer (N: P: K = 21:8:16) at a rate of 3 g per pot per application. In April 2023, one year after planting, root length, plant height, and dry weight of *C. equisetifolia* seedlings above and below ground were determined. Plant height and root length were measured directly using a tape measure, where plant height is the height from the point of separation of roots and stems to the highest point of the plant, and root length is the length from the point of separation of roots and stems to the point where the root system is longest. The seedlings were cut into aboveground and belowground parts at the point of separation of roots and stems, then they were fixed at 120°C for 15 min and dried at 80°C until constant weight, and then their weights were measured, which were the dry weights of above and below ground.

### 2.3 Determination of basic soil physicochemical characteristics and enzyme activities

In this research, key soil physicochemical characteristics were identified, including soil cation exchange capacity (CEC), available nitrogen (AN), available phosphorus (AP), and available potassium (AK), using established methodologies in accordance with Wang et al. (2023). CEC was measured using the neutral ammonium acetate method, which involved treating the soil with a solution of 1 mol/L neutral ammonium acetate and then obtaining a boric acid solution containing ammonia by distillation, and hydrochloric acid was then used for titration to convert the result into CEC. The AN content was determined using an alkali hydrolysis diffusion method. Specifically, 1 mol/L NaOH solution was used for leaching, filtration, and the filtrate was subsequently filtered with hydrochloric acid to determine AN content. The AP content was determined using NaHCO_3_ extraction-molybdenum antimony colorimetric method. Specifically, 0.5 mol/L NaHCO_3_ solution was used for extraction, which was then filtered and the filtrate was added to a mixture of molybdenum antimony colorimetric agent before the absorbance was measured at 880 nm and subsequently converted to AP content. The AK content was determined using the ammonium acetate extraction-flame photometer method. Specifically, 1 mol/L neutral ammonium acetate was utilized for extraction, followed by filtration and determination of the resulting filtrate via a Single-Channel M410 Flame Photometer (Cole-Parmer, USA).

Enzyme Linked Immunosorbent Assay Kit (Shanghai Preferred Biotechnology Co., Ltd.) was used in this study to determine the activities of soil enzymes that included urease, polyphenol oxidase, protease, cellulase, acid phosphatase, superoxide dismutase, sucrase, catalase, peroxidase. Extraction of soil enzymes was carried out according to the instructions provided by the enzyme immunoassay kit, using 1 g of fresh soil. The absorbance was subsequently measured using a multifunctional enzyme labeling instrument (BioTek Synergy2 Gene 5, Vermont, USA) to determine the soil enzyme activity, which was expressed as U/g. The absorbance of urease, acid phosphatase, protease, sucrase, cellulase, superoxide dismutase, catalase, peroxidase, and polyphenol oxidase was measured at various wavelengths including 630, 660, 680, 540, 540, 560, 240, 470, and 430 nm, respectively. Three replications were established independently for each treatment.

### 2.4 Determination of soil microbial biomass carbon, nitrogen and respiration intensity

Soil microbial biomass carbon, nitrogen and respiration intensity were quantified using the approach outlined by Zhang et al. (2021). Soil microbial biomass carbon and nitrogen were measured by a chloroform fumigation extraction technique with three independent replicates per sample to ensure accuracy. The microbial biomass carbon was calculated as Ec/Kc, where Ec represents the fumigated organic carbon minus unfumigated organic carbon, and Kc is a value of 0.38. The microbial biomass nitrogen was determined by calculating with formula: (fumigated soil total nitrogen – unfumigated soil total nitrogen)/0.54. Soil microbial respiration intensity (mg CO_2_/kg·h) was measured as the amount of CO_2_ released per kilogram of soil per hour using the alkali absorption method.

### 2.5 *q*RT-PCR analysis of soil bacterial, fungal and actinomycete quantity

Extraction of soil DNA was performed using the Bio-Fast Soil Genomic DNA Extraction Kit (BioFlux, Hangzhou, China), and fresh soil was incorporated at a dose of 0.5 g. DNA detection was achieved by 1% agarose gel electrophoresis followed by purification with the Gel Recovery Kit (TianGen Biotch. Co., Ltd.). For quantifying bacteria, fungi, and actinomycetes, the *q*RT-PCR protocol described in Ye et al. (2023a) was applied. For quantitative analysis of bacteria, specific primers were used. These primers were F27 and R1492, the sequence of which was 5′-AGAGTTTGATCMTGCCTCAG-3′ and 5′-TACHHYTACCTTGTTACGACTT-3′, respectively. The PCR reaction program involved pre-denaturation at 94°C for 4 min, followed by 1 min at 94°C, 1 min at 55°C, and 1 min at 72°C. The program also involved 30 cycles. Primers for quantitative analysis of fungi were 5.8S with sequence 5′-CGCTGCGTTCTTCATCG-3′ and ITSIF with sequence 5′-CTTGGTCATTTAGAGGAAGTAA-3′. The PCR reaction program consisted of pre-denaturing the samples at 95°C for 5 s, denaturing at 94°C for 30 s, annealing at 53°C for 30 s, and extending at 72°C for 30 s with a total of 40 cycles. Two primers have been developed for quantitative analysis of actinomycetes. The first primer, act920f, has the sequence 5′-TACGGCCGCAAGGCTA-3′ and the second primer, act1200r, has the sequence 5′-TCRTCCCCACCTTCCTCCG-3′. The PCR reaction program was composed of pre-denaturation at 95°C for 10 min, followed by 95°C for 15 s, 65°C for 30 s, 72°C for 15 s, and 40 cycles. 

### 2.6 Soil metagenome sequencing

#### 2.6.1 Metagenomic sequencing analysis

Total DNA was extracted using the instructions provided in the Bio-Fast Soil Genomic DNA Extraction Kit at a soil dose of 0.5 g. The extracted DNA was subjected to electrophoresis on a 1% agarose gel to examine its integrity. Quantification of DNA concentrations was achieved using the Qubit^®^ dsDNA Assay Kit on a Qubit^®^ 2.0 Flurometer (Life Technologies, CA, USA). When the OD value falls within the range of 1.8 to 2.0 and the DNA content exceeds 1μg, it is deemed suitable for the construction of libraries.

The approach to building a library was summarized below. An input amount of 1 μg DNA per sample was utilized as for DNA sample preparation. The creation of sequencing libraries was carried out through NEB Next^®^ Ultra™ DNA Library Prep Kit for Illumina (NEB, Texas, USA), according to the manufacturer’s instructions and index codes were attached in order to attribute sequences to each sample. Sonication was used to fragment the DNA sample to a size of about 350 bp, and then DNA fragments underwent end-polishing, A-tail, and ligation with the full-length adaptor for Illumina sequencing. This was followed by PCR amplification. Purification of PCR products was performed using AMPure XP system (Beckman Coulter, Beverly, USA), and library size distribution and quantity were analyzed using an Agilent2100 Bioanalyzer and real-time PCR (Agilent, California, USA). A brief description of the sequencing method was provided below. After index coding the samples, they were clustered on the cBot Cluster Generation System. Following the cluster generation process, library preparations were sequenced on an Illumina NovaSeq platform and paired-end reads were generated (Wang et al., 2022c).

#### 2.6.2 Bioinformatics analysis

Pre-processing of sequencing involves various steps (Karlsson et al., 2012; Scher et al., 2013). Fastp software was used for Raw Data quality control. Software parameters were set by default to process Raw Data obtained from Illumina HiSeq sequencing platform. Clean Data was obtained for subsequent analysis. If the sample was contaminated in the sample, a comparison with the host database was made to filter out host-derived reads. Bowtie2 was selected as the aligner, and the parameters were set to –sensitive, -I 200, -x 400.

After the above pretreatment, Clean Data was obtained and then analyzed for metagenome assembly using the MEGAHIT assembly software (Version v1.2.9) (Karlsson et al., 2012; Zeller et al., 2014). The Assembly parameters consisted of a minimum K of 35, a maximum K of 95 with a step of 20, and a minimum contig length of 500 base pairs. The clean data of each sample after quality control was compared to the contigs of each sample after assembly using Bowtie2 software (Version 2.3.4) to obtain PE reads that were not used during assembly. To compare assemblies, the following parameters were used: -I 200, -x 400. Unused reads from each sample were combined to form a mixed assembly. The assembly parameters used for the mixed assembly were the same as to those used for a single sample.

Based on contigs (≥ 500bp) of each sample and mixed assembly, MetaGeneMark software (Version 3.38) was used to predict ORF (Open Reading Frame), and default parameters were used (Fu et al., 2012; Cotillard et al., 2013; Buchfink et al., 2015). Based on the predicted results, predicted genes with a length of less than 100 nucleotides were screened. Combine ORF prediction results from all samples and mixed assembly, and use CD-HIT software (Version 4.8.1) for redundancy removal, generating the initial gene catalogue consisting of non-redundant nucleic acid sequences. By default, 95% identity value and 90% coverage value were used for clustering, and the representative sequence was selected based on the longest sequence. The parameters have been set to -c 0.95, -g 0, -AS 0.9, -g 1, -d 0. The Clean Data of each sample was compared to the original gene catalogue using Bowtie2 software (Version 2.3.4), and the number of reads that the gene was compared in each sample was calculated. A sensitive approach and end-to-end alignment were used, with a maximum insertion size of 200 and a minimum overlap length of 400, to identify genes. The supporting genes below 2 in each sample were filtered to obtain a gene catalogue (Unigenes) for subsequent analysis. The abundance of information for each gene in each sample was then calculated based on the number of reads and gene length compared. In addition, core and pan-gene dilution curves were further analyzed to assess stability of the assayed samples (Xu et al., 2023).

DIAMOND software (Version v0.9.25) was used to compare unigenes sequences with those of bacteria, fungi, archaea, and viruses, which were extracted from the NCBI NR database. The BLastp and EVALue parameters were set to ≤ 1E-5 to achieve an accurate comparison between the two datasets (Ondov et al., 2011; Karlsson et al., 2013). Because each sequence may generate multiple comparison results, multiple species classification information can be obtained. To ensure that the sequence has biological significance, the MEGAN software (Version 6.21.5) LCA algorithm was employed to obtain the final species annotation information for the sequence. Using the result of the LCA annotation and gene abundance table, the abundance of information for each sample at each classification level (phylum and genus) was obtained. The abundance of a species within a sample was determined by summing up the gene abundance of the species. Using the LCA annotation results and the gene abundance table, the gene number table of each taxonomic sample (phylum, compendium and genus) was generated. The gene number of a species in a sample was equal to the gene number of the annotated species except in cases where gene abundance equaled zero. Alpha diversity reflects the species abundance and species diversity of individual samples, and is measured by a variety of metrics, including richness, Chao1, Shannon, and Simpson. Of these, richness and Chao1 are used to measure species abundance, while Shannon and Simpson are used to measure species diversity (Willis, 2019). In this study, QIIME2 software (Version 2021.4) was used to assess the above indexes.β-diversity can be used to assess the differences in species diversity between samples. In this study, the bray curtis algorithm was used to calculate the distance between samples and analyze the β-diversity between samples (Xiao et al., 2021). Based on this, the OPLS-DA model was further constructed in this study (Zhang et al., 2023c). After the goodness-of-fit and predictability of the model were assessed to be qualified, key microorganisms were screened by the important projected value (VIP value) of the model indexes (Jia et al., 2023b; Zhang et al., 2023b). In addition, XGBoost machine deep learning was applied to model construction to obtain the importance of key microorganisms in distinguishing different samples (Chen et al., 2020; Khan et al., 2020).

### 2.7 *q*RT-PCR analysis of *Actinoallomurus, Actinomadura*, and *Mycobacterium* quantity in soil

Extraction of soil DNA was achieved using the Bio-Fast Soil Genomic DNA Extraction Kit, which involved the use of 0.5 g of fresh soil. DNA was detected for quality by 1% agarose gel electrophoresis and purified by the Gel Recovery Kit for *q*RT-PCR quantification of *Actinoallomurus* sp, *Actinomadura* sp, and *Mycobacterium* sp. The primers used for quantitative analysis of *Actinoallomurus* were 5′-GAAGAAGCGCCGGGCTAACTA-3′ and 5′-ACGAGCTCTTTACGCCCAAT-3′. The primers for quantitative analysis of *Actinomadura* were 5′-CACACTGGGACTGAGACACG-3′ and 5′-TTCGTCGGGTGCTGAAAGAGG-3′. The primers for quantitative analysis of *Mycobacterium* were 5′-GGCGTGCTTAACACATGCAA-3′ and 5′-AGGCTTATCCCGAAGTGCAG-3′. The PCR reaction programs encompassed a pre-denaturation phase at 95°C for 5 min, followed by 15 s at 95°C, 30 s at 60°C, 15 s at 72°C, and 30 cycles.

### 2.8 Statistical analysis

Excel 2017 software was used to pre-process the raw data, including the calculation of statistical measures such as mean and standard deviation. SPSS 21.0 statistical software was used to perform T-text tests, correlation analysis, and the creation of both conventional bar graphs and stacked graphs. Rstudio software (R version 4.2.3) was used to produce box plots (R library was gghalves 0.1.4), correlation matrix analysis (R libraries was linkET 0.0.7.1), principal component plots (R library was ggbiplot 0.55), heat maps (R library was pheatmap 1.0.12), orthogonal partial least squares-discriminate analysis model (OPLS-DA, package used for this was ropls and mixOmics), XGboost (R libraries was ggplot2 version 3.4.0), redundancy analysis (R library was vegan 2.6.4), and interaction network diagrams (R libraries was linkET 0.0.7.1).

## 3 Results

### 3.1 Effect of continuous planting on the growth of *C. equisetifolia* seedlings

**Figure 1:**
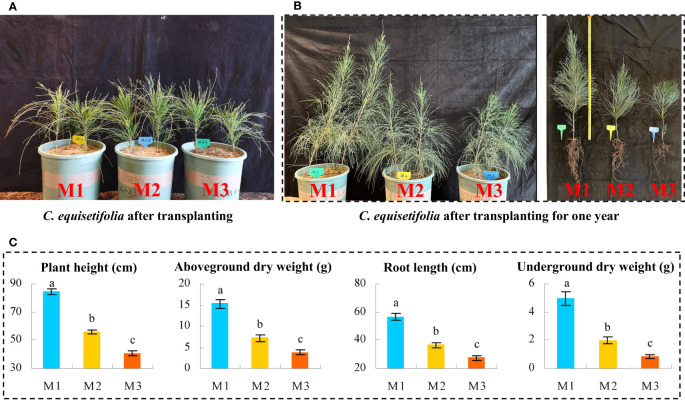
Effect of continuous planting on *Casuarina equisetifolia* seedlings growth. M1: First planting; M2: Second continuous planting; M3: Third continuous planting; **(A)** The photograph of *Casuarina equisetifolia* seedlings freshly transplanted in pots; **(B)** The photograph of *Casuarina equisetifolia* seedlings transplanted in pots one year after transplanting and the photograph of single plants; **(C)** Analysis of basal growth indexes of *Casuarina equisetifolia* seedlings after transplanting for one year; The data in the figure were mean value ± SD, and the significance of differences was tested by T-test. Different lowercase letters indicate significant differences between treatments at the *p* < 0.05 level.

In this study, we collected the root soil of *C. equisetifolia* trees with different numbers of continuous planting and replanted the *C. equisetifolia* seedlings (**Figure 1A**) to analyze the effect of continuous plantings on the seedlings growth, and the results showed (**Figure 1B**) that after continuous planting, the root soil of *C. equisetifolia* trees did have significant effects on its growth, with shorter height, shorter root length, and fewer roots. Further determination of morphological traits revealed (**Figure 1C**) that plant height, root length, above-ground dry weight, and below-ground dry weight of *C. equisetifolia* significantly decreased with increasing number of continuous plantings (M1 to M3), as evidenced by a decrease from 84.26 to 40.74 cm, 56.59 to 27.34 cm, 15.26 to 3.76 g, and 4.96 to 0.82 g, respectively. It can be seen that continuous planting does hider growth, and the extent of its effect increases with the number of continuous plantings.

### 3.2 Physicochemical property of rhizosphere soil of *C. equisetifolia* trees

**Figure 2:**
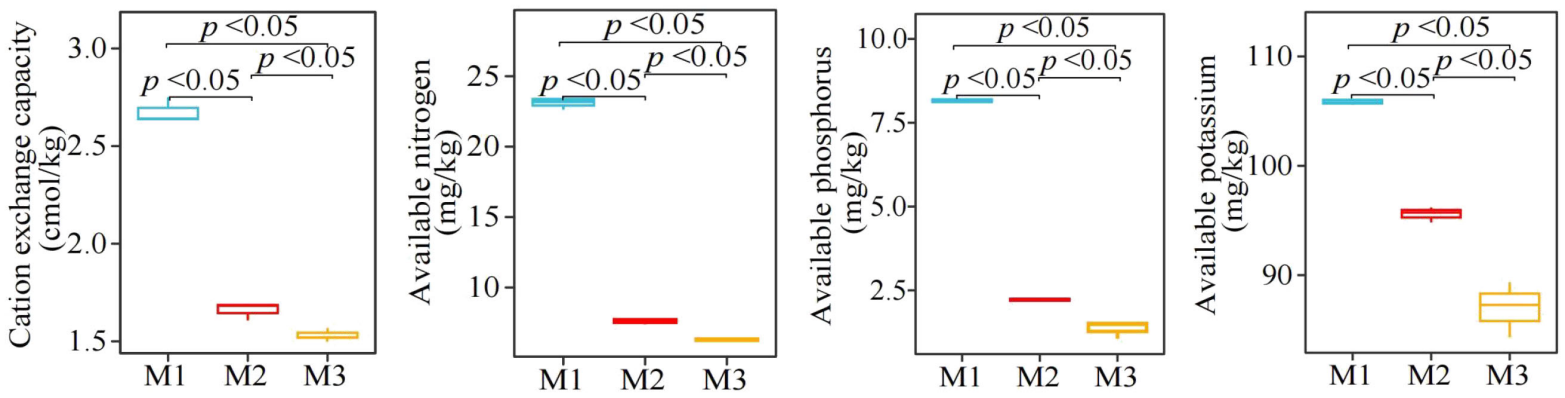
Effect of continuous planting on the physicochemical property of *Casuarina equisetifolia* trees rhizosphere soil. M1: First planting; M2: Second continuous planting; M3: Third continuous planting; The data in the figure were mean value ± SD, and the significance of differences was tested by T-test.

Analysis of physicochemical property of *C. equisetifolia* tree rhizosphere soil showed (**Figure 2**) that with increasing number of continuously planting *C. equisetifolia* trees (M1-M3), the CEC, AN, AP, and AK contents of the rhizosphere soil decreased significantly from 2.68 to 1.54 cmol/kg, 23.13 to 6.29 mg/kg, 8.16 to 1.36 mg/kg, 105.89 to 86.98 mg/kg, respectively. It can be seen that continuous planting reduced the available nutrient content and ion exchange capacity of the rhizosphere soil of *C. equisetifolia* trees.

### 3.3 Rhizosphere soil enzyme activities of *C. equisetifolia* trees

**Figure 3:**
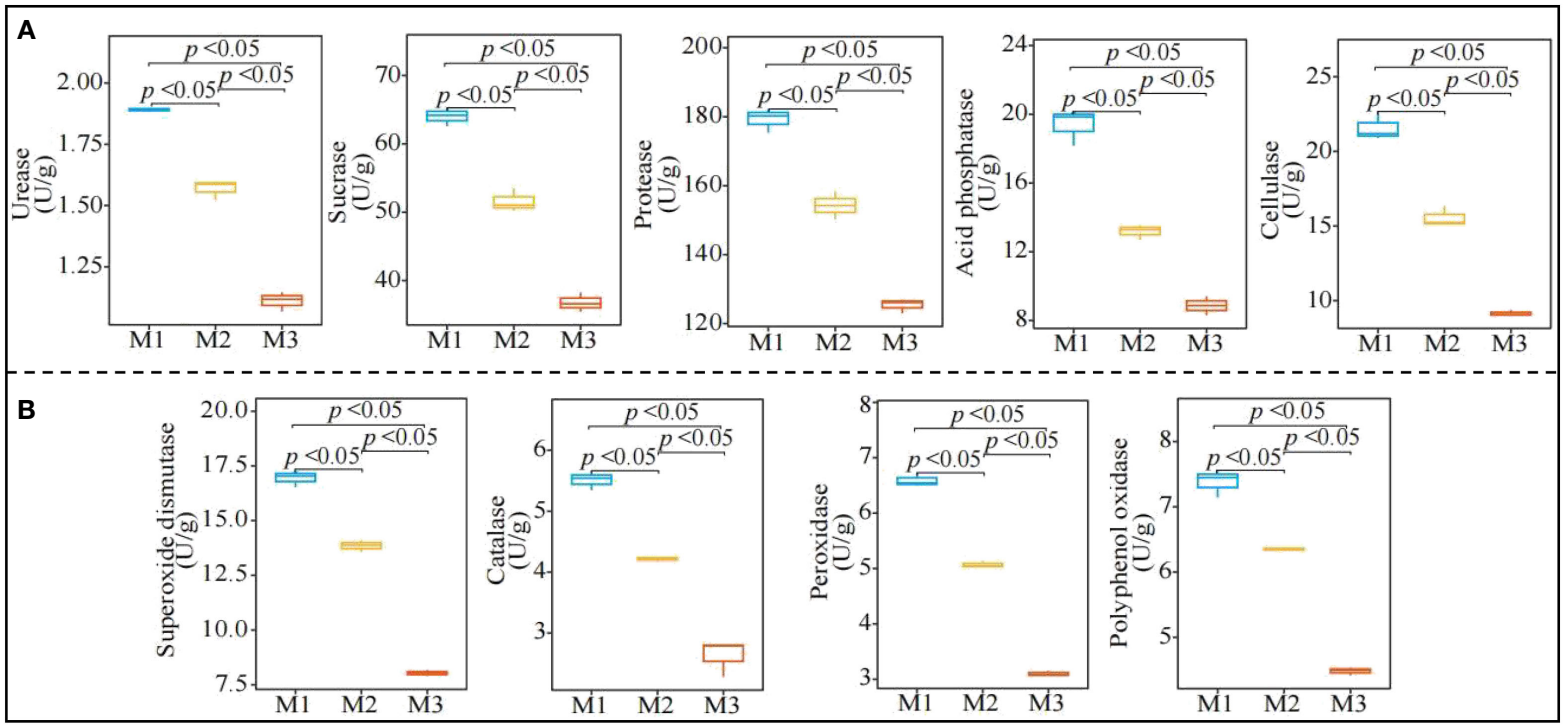
Effect of continuous planting on the enzyme activities of *Casuarina equisetifolia* trees rhizosphere soil. M1: First planting; M2: Second continuous planting; M3: Third continuous planting; **(A)** Effect of continuous planting on nutrient cycling-related enzyme activities in the *Casuarina equisetifolia* trees rhizosphere soil; **(B)** Effect of continuous planting on resistance-related enzyme activities in the *Casuarina equisetifolia* trees rhizosphere soil; The data in the figure were mean value ± SD, and the significance of differences was tested by T-test.

In this study, it was found (**Figure 3A**) that with increasing number of continuous plantings of *C. equisetifolia* trees (M1-M3), the activity of rhizosphere soil urease, sucrase, protease, acid phosphatase, and cellulase showed a significant decreasing trend from 1.89 to 1.11 U/mg, 64.04 to 36.77 U/mg, 179.27 to 125.33 U/mg and 21.58 to 9.16 U/mg, respectively. The results of the analysis of soil resistance-related enzyme activities (**Figure 3B**) showed that with the increasing number of continuously planting *C. equisetifolia* trees (M1-M3), the activity of rhizosphere soil superoxide dismutase, catalase, peroxidase, and polyphenol oxidase showed a decreasing trend, from 16.94 to 8.03 U/mg, 5.51 to 2.63 U/mg, 6.59 to 3.10 U/mg and 7.38 to 4.48 U/mg, respectively. It can be seen that continuous planting significantly reduced the activities of rhizosphere soil nutrient cycling-related enzymes and resistance-related enzymes of *C. equisetifolia* trees.

### 3.4 Rhizosphere soil microbial quantity and physiological indexes of *C. equisetifolia* trees

**Figure 4:**
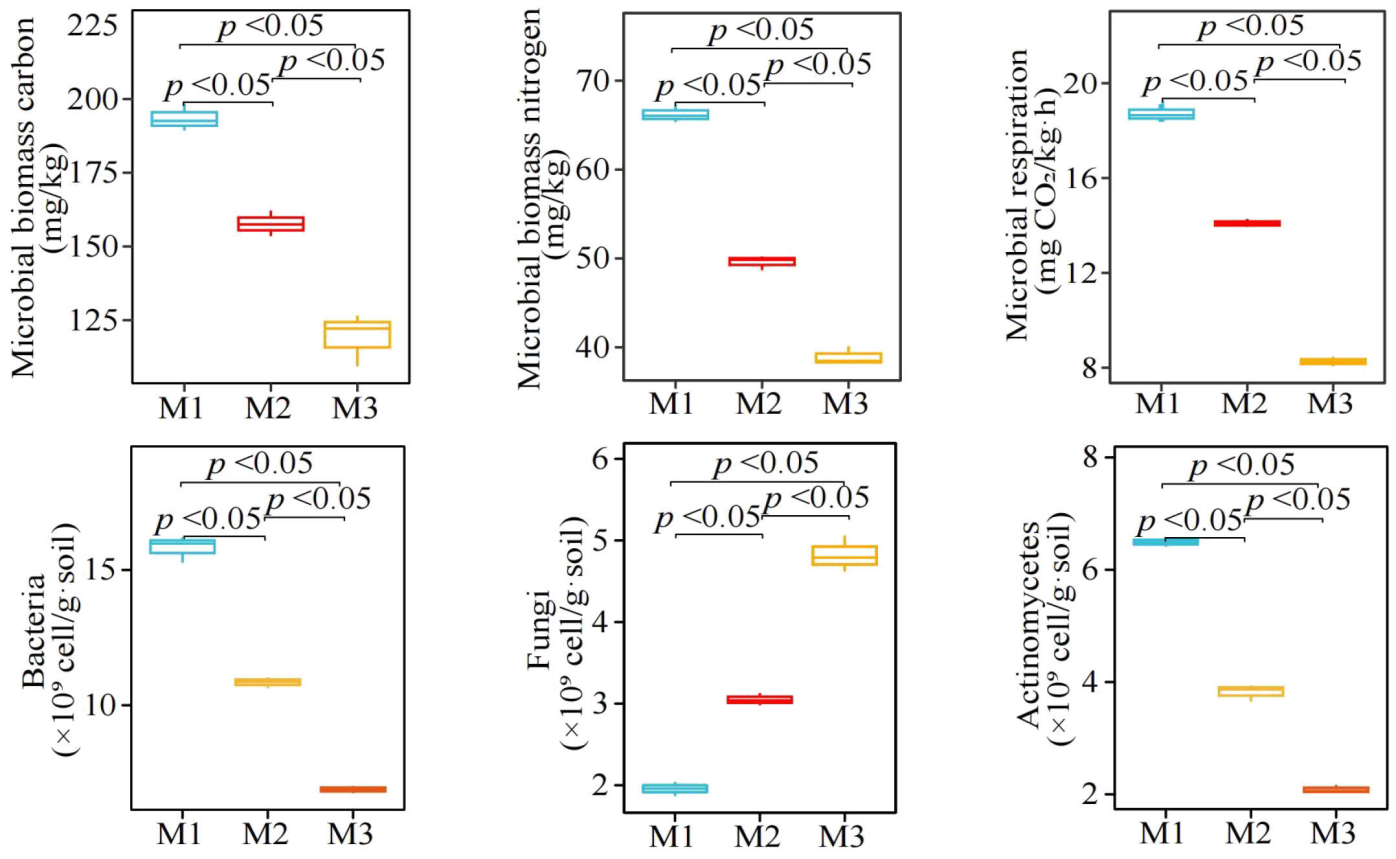
Effect of continuous planting on the quantity of microorganisms and physiological characteristics in *Casuarina equisetifolia* trees rhizosphere soil. M1: First planting; M2: Second continuous planting; M3: Third continuous planting; The data in the figure were mean value ± SD, and the significance of differences was tested by T-test. Different lowercase letters indicate significant differences between treatments at the *p* < 0.05 level.

In this study, it was found (**Figure 4**) that with the increase in the number of continuously planting *C. equisetifolia* trees (M1-M3), the rhizosphere soil microbial biomass carbon decreased from 1983.46 to 119.35 mg/kg, and microbial biomass nitrogen decreased from 66.22 to 38.92 mg/kg, and microbial respiration from 18.71 to 8.26 mg CO_2_/kg·h. Secondly, with the increase in the number of continuously planting *C. equisetifolia* trees (M1-M3), the quantity of bacteria and actinomycetes in the rhizosphere soil decreased significantly, i.e., from 15.82 to 6.89×10^9^ cell/g·soil and 6.49 to 2.09 ×10^9^ cell/g·soil, respectively, whereas the quantity of fungi showed an upward trend, i.e., from 1.96 to 4.82 × 10^9^ cell/g·soil. It can be seen that continuous planting significantly affected the rhizosphere soil microbial community structure of *C. equisetifolia* trees.

### 3.5 Correlation matrix analysis of soil physicochemical property, soil enzymes, and soil microorganisms

**Figure 5:**
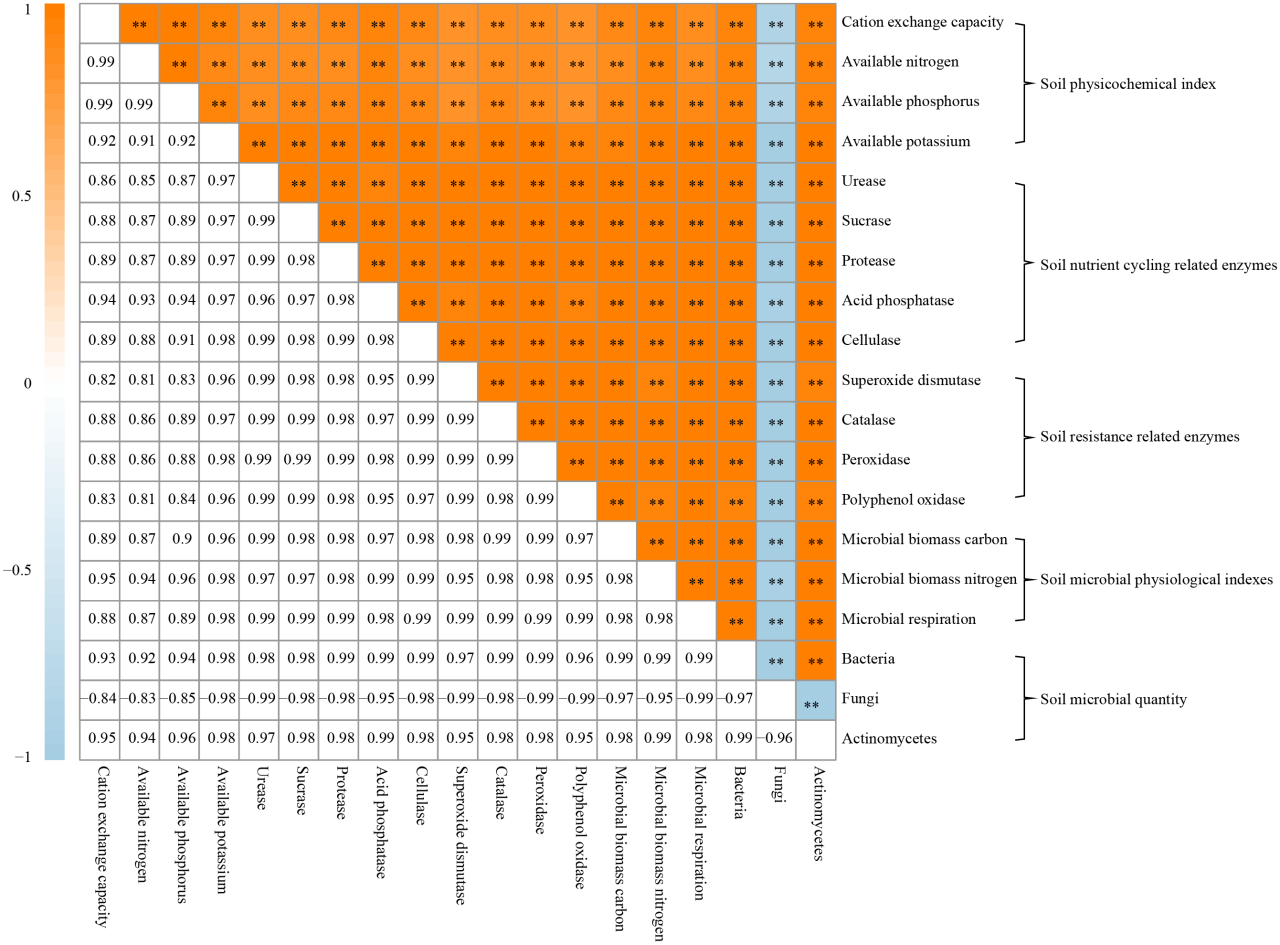
Correlation matrix analysis between physicochemical indexes, soil enzymes, and soil microorganisms in *Casuarina equisetifolia* trees rhizosphere soil. **indicates differences between samples at the *p* < 0.01 level.

On the basis of the previous study, this study further analyzed the relationships between rhizosphere soil physicochemical property, soil enzymes and soil microorganisms of *C. equisetifolia* trees, and the results showed (**Figure 5**) that there were significant positive correlations between soil physicochemical property, soil nutrient-cycling related enzyme activities, soil resistance-related enzyme activities, soil microbial physiological property, bacterial quantity, actinomycete quantity with each other, and a significant negative correlation with soil fungi quantity. Continuous planting has altered community structure and quantity of soil microorganisms in the rhizosphere zone, reduced soil nutrient cycling and resistance-related enzyme activities, and reduced soil available nutrients, which in turn affected tree growth.

### 3.6 Metagenomic analysis of soil microorganisms in the *C. equisetifolia* tree rhizosphere

**Figure 6:**
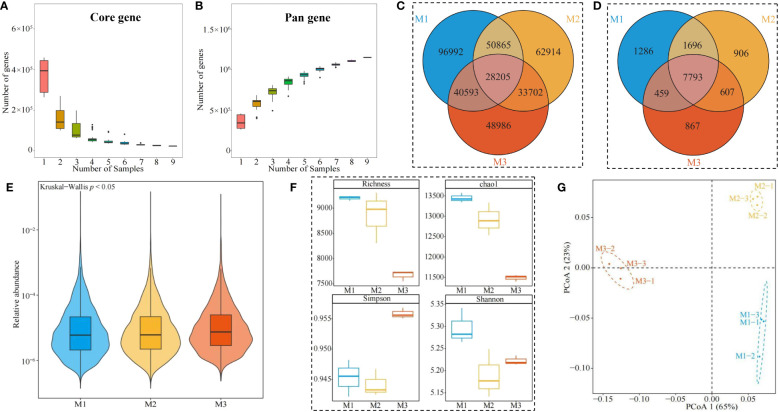
Soil microbial metagenome analysis. M1: First planting; M2: Second continuous planting; M3: Third continuous planting; **(A)** Core gene dilution curve of *Casuarina equisetifolia* trees rhizosphere soil; **(B)** Pan gene dilution curve of *Casuarina equisetifolia* trees rhizosphere soil; **(C)** Venn diagram analysis of genes in the *Casuarina equisetifolia* trees rhizosphere soil; **(D)** Venn diagram analysis of microorganisms in the *Casuarina equisetifolia* trees rhizosphere soil; **(E)** Analysis of variance of total abundance of microorganisms in the *Casuarina equisetifolia* trees rhizosphere soil; **(F)** Analysis of α-diversity index of microorganisms in the *Casuarina equisetifolia* trees rhizosphere soil; **(G)** PCoA plot of β-diversity analysis of microorganisms in the *Casuarina equisetifolia* trees rhizosphere soil.

The rhizosphere soil of *C. equisetifolia* trees with different numbers of continuous planting was analyzed by sequencing using Illumina, and a total of 64.86 G of Clean Data was obtained from nine samples (**Table S1**). Clean Data was assembled and analyzed, and a total of 2,178,435 contigs were obtained with a total length of 1,878,521,359 bp (**Table S2**). The ORF (Open Reading Frame) prediction of contigs was performed, and a total of 2,202,069 genes were obtained, with the total length of the genes in the gene catalogue being 1,010.4 Mbp, and the average length of the genes was 458.84 bp (**Table S3**). Analysis of the dilution curves of core genes in this study showed (**Figure 6A**) that core genes decreased with the increase in the number of samples, and when the number of samples reached 7, the number of core genes stabilized. Analysis of the dilution curve of pan genes showed (**Figure 6B**) that pan genes increased with the number of samples, and the number of pan genes stabilized when the number of samples reached 7. It can be seen that the metagenomic sequencing results of soil samples in this study can basically reflect all microorganisms in the soil and the sequencing results can be used for further analysis.

Accordingly, in this study, we further performed a comparative analysis of rhizosphere soil genes of *C. equisetifolia* trees with different numbers of continuous planting, and the results showed (**Figure 6C**) that 28,205 genes were common to rhizosphere soils of M1, M2, and M3, of which 96,992 genes were unique to M1, 62,914 genes were unique to M2, and 48,986 genes were unique to M3. Species annotation with detected genes and comparative analysis revealed (**Figure 6D**) that there were a total of 7,793 microbial species in rhizosphere soils of M1, M2, and M3, of which 1,286 were M1-specific, 906 were M2-specific, and 867 were M3-specific. Analysis of the differences in the overall abundance of soil microorganisms in the *C. equisetifolia* tree rhizosphere with different numbers of continuous planting showed (**Figure 6E**) that there was a significant difference in microbial abundance between M1, M2, and M3 (*p* < 0.05). Further analysis of the α-diversity indexes of microorganisms in the rhizosphere soil with different numbers of continuous planting revealed (**Figure 6F**) that the richness and Chao1 indexes of soil microorganisms showed a decreasing trend as the number of continuous planting increased (M1~M3), while the Simpson and Shannon indexes showed a decreasing and then increasing trend. The PcoA analysis of β-diversity showed (**Figure 6G**) that the two principal components, which can effectively distinguish M1, M2, and M3 in different regions, had an overall contribution of 88%. It can be seen that continuous planting led to a decrease in the microbial richness of the rhizosphere soil, and there were significant differences in the diversity of microbial communities in the rhizosphere soil with different numbers of continuous planting.

**Figure 7:**
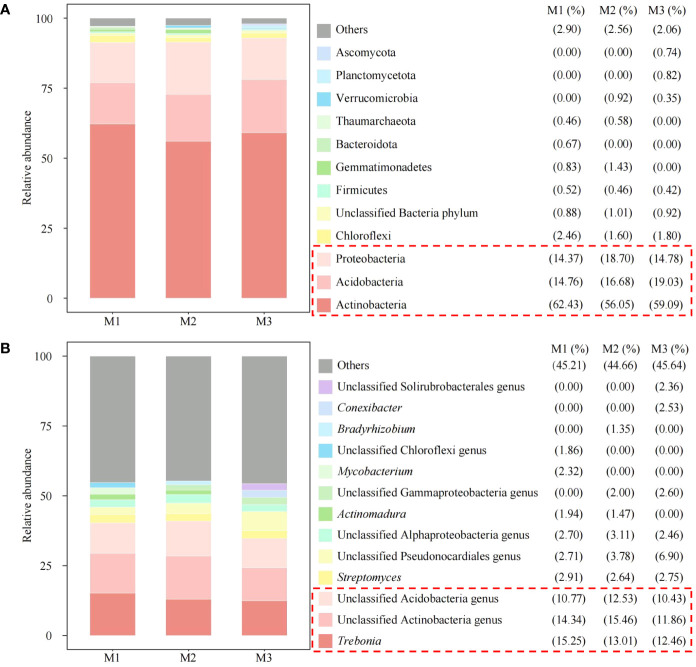
Abundance analysis of soil microorganisms at phylum level and genus level. M1: First planting; M2: Second continuous planting; M3: Third continuous planting; **(A)** Abundance analysis of soil microorganisms at phylum level; **(B)** Abundance analysis of soil microorganisms at genus level; Stack plots were created with the top ten microbes in each sample in terms of abundance, with the rest categorized as others.

On this basis, this study further analyzed the microbial community structure of rhizosphere soil under continuous planting of *C. equisetifolia* trees and found that, in terms of phylum classification, the top 3 dominant microorganisms in rhizosphere soil with different numbers of continuous planting were Proteobacteria, Acidobacteria, Actinobacteria, respectively (**Figure 7A**). Classified at the genus level, the top 3 dominant microorganisms in rhizosphere soil with different numbers of continuous planting were unclassified Acidobacteria genus, unclassified Actinobacteria genus, and *Trebonia.* The above three microbial genera accounted for 10.77%, 14.34%, and 15.25% in M1, 12.53%, 15.46%, and 13.01% in M2, and 10.43%, 11.86%, and 12.46% in M3, respectively (**Figure 7B**). It can be seen that the dominant microbial quantity in rhizosphere soil with different numbers of continuous planting was more similar, but with some differences in abundance.

### 3.7 Screening and classification analysis of key differential microorganisms

**Figure 8:**
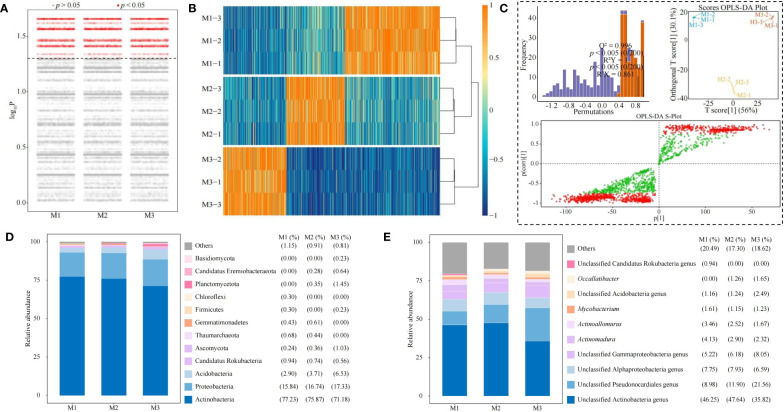
Screening and analysis of key soil microorganisms. M1: First planting; M2: Second continuous planting; M3: Third continuous planting; **(A)** Kruskal test for differences in microbial abundance between groups; **(B)** Wilcoxon rank sum test to obtain a heat map of 2,115 microbes with significant differences between groups; **(C)** Screening of key differential microorganisms based on intergroup OPLS-DA modeling; **(D)** Abundance analysis of key differential microorganisms at the phylum level; **(E)** Abundance analysis of key differential microorganisms at the genus level; Microbial abundance at the phylum and genus level was analyzed, and stacked plots were made with the top ten in terms of abundance percentage in each sample, with the rest categorized as others.

Based on the previous analysis, this study further analyzed differences in microbial abundance between groups (M1, M2, and M3) and showed (**Figure 8A**) that a total of 2,115 microbial species with significant differences were obtained (*p* < 0.05). Heat map analysis showed (**Figure 8B**) that out of 2,115 microorganisms, 928 microorganisms significantly differentiated M1, 573 microorganisms significantly differentiated M2, and 614 microorganisms significantly differentiated M3. It was seen that there were significant differences in the abundance of microorganisms in rhizosphere soil of *C. equisetifolia* trees with different numbers of continuous planting.

Accordingly, this study further constructed the OPLS-DA model among M1, M2, and M3 and tested the model, and the results showed (**Figure 8C**) that the constructed OPLS-DA model, with its goodness-of-fit value of R^2^Y = 1, *p* < 0.005, and predictability Q^2 = ^0.996, *p* < 0.005, after permutation test; the score plots showed that different samples could be effectively distinguished in different regions; S-plot showed that the total number of key microorganisms with VIP value > 1 was 1,254. The results showed (**Figure 8D**) that at the phylum level, the top 10 phylums in terms of microbial abundance in the rhizosphere soil of *C. equisetifolia* trees with different numbers of continuous planting were mainly Basidiomycota, Candidatus Eremiobacteraeota, Planctomycetota, Chloroflexi, Firmicutes, Gemmatimonadetes, Thaumarchaeota, Ascomycota, Candidatus Rokubacteria, Acidobacteria, Proteobacteria, and Actinobacteria, and especially Actinobacteria had the largest proportion. At the genus level, the top 10 genera in terms of microbial abundance in the rhizosphere soil with different numbers of continuous planting were mainly unclassified Candidatus Rokubacteria genus, *Occallatibacter*, unclassified Acidobacteria genus, *Mycobacterium*, *Actinoallomurus*, *Actinomadura*, unclassified Gammaproteobacteria genus, unclassified Alphaproteobacteria genus, unclassified Pseudonocardiales genus, unclassified Actinobacteria genus, and especially Actinobacteria genus had the largest proportion (**Figure 8E**). It can be seen that continuous planting resulted in significant changes in the community abundance of soil microorganisms in *C. equisetifolia* tree rhizosphere.

### 3.8 Abundance analysis of key differential microorganisms and their importance evaluation

**Figure 9:**
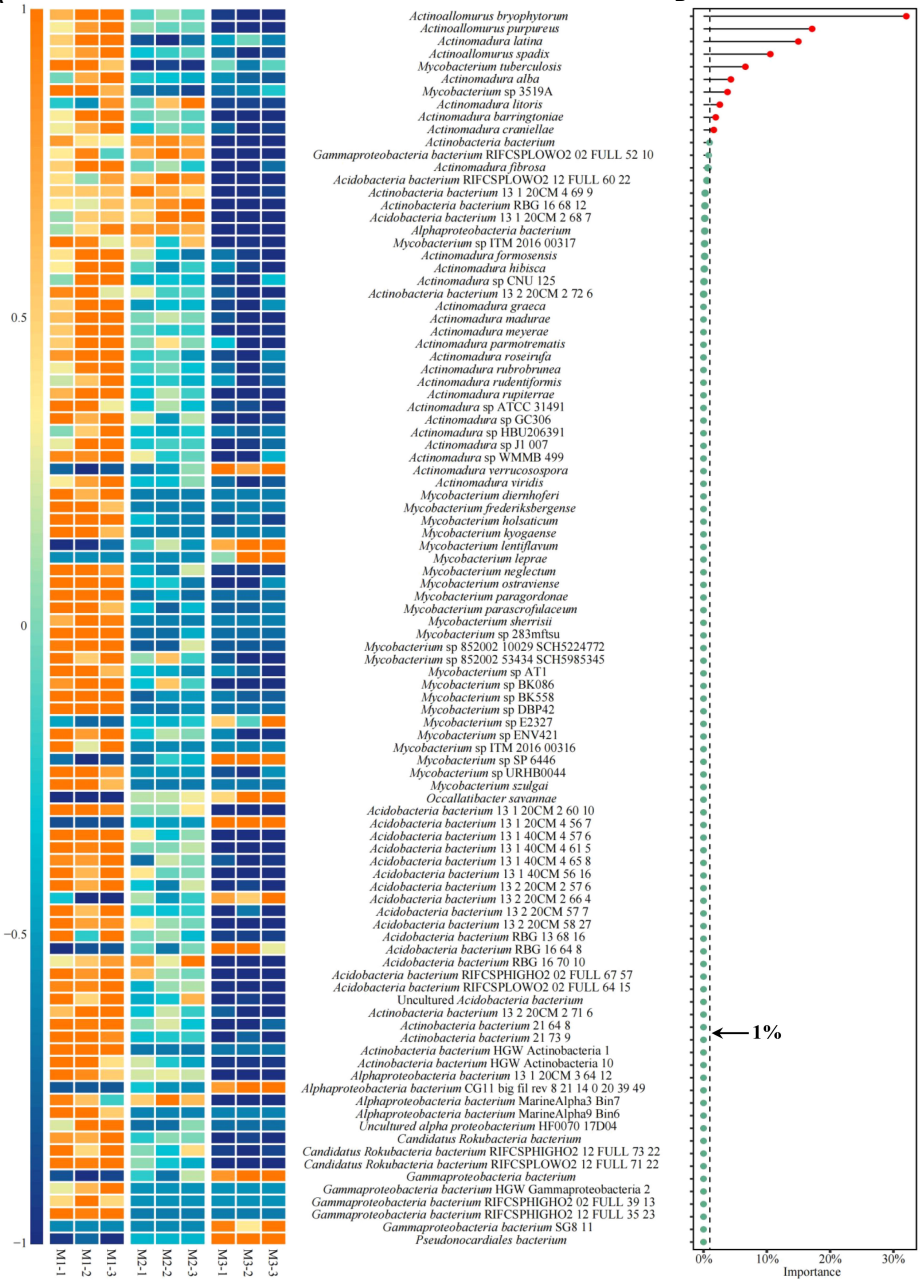
Abundance analysis of key microorganisms and their importance evaluation. M1: First planting; M2: Second continuous planting; M3: Third continuous planting; **(A)** Heat map analysis of 98 key microorganisms; **(B)** Feature importance of 98 microorganisms in distinguishing M1, M2, and M3 using XGBoost machine deep learning.

**Figure 10:**
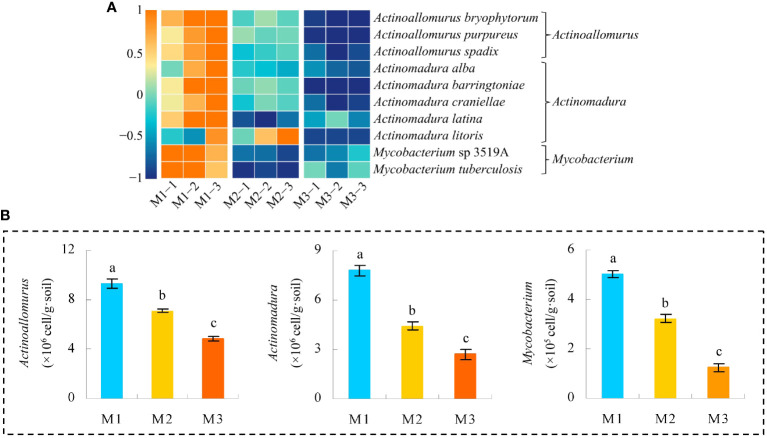
Abundance analysis of 10 characteristic microorganisms and quantitative validation by *q*RT-PCR. M1: First planting; M2: Second continuous planting; M3: Third continuous planting; **(A)** Heat map analysis of abundance of 10 characteristic microorganisms; **(B)** Quantitative *q*RT-PCR analysis of microbial quantity of *Actinoallomurus*, *Actinomadura*, and *Mycobacterium* in soil; The data in the figure were mean value ± SD, and the significance of differences was tested by T-test. Different lowercase letters indicate significant differences between treatments at the *p* < 0.05 level.

Based on the previous analysis, this study found that the key microorganisms that distinguished M1, M2, and M3 were mainly from 10 genera. The microbial abundance of the 10 genera was further analyzed in this study, and the results showed (**Figure 9A**) that a total of 98 microorganisms were from these 10 genera, of which 13 showed an increasing trend in abundance and 85 showed a decreasing trend as the number of continuously planted *C. equisetifolia* trees increased. Accordingly, in this study, XGBoost machine deep learning was used to screen microorganisms of feature importance, and the results showed (**Figure 1S**) that 98 microorganisms constructed the XGBoost model with 100% confusion matrix accuracy, and all ROC curves for the differentiation of M1, M2, and M3 also reached 100% accuracy. It can be seen that 98 microorganisms were able to effectively differentiate between the different samples. The feature importance values of 98 microorganisms were further derived from the XGBoost model, and the results showed (**Figure 9B**) that 23 microorganisms had feature importance values distributed between 0.02% and 32.04%, and 75 microorganisms had feature importance values of zero. Secondly, among the 23 microorganisms mentioned above, only 10 microorganisms had feature importance values greater than 1%, namely *Actinoallomurus bryophytorum* (32.04%), *Actinoallomurus purpureus* (17.15%), *Actinomadura latina* (14.98%), *Actinoallomurus spadix* (10.56%), *Mycobacterium tuberculosis* (6.62%), *Actinomadura alba* (4.32%), *Mycobacterium* sp 3519A (3.79%), *Actinomadura litoris* (2.57%), *Actinomadura barringtoniae* (1.93%), *Actinomadura craniellae* (1.62%), mainly from three genera: *Actinoallomurus*, *Actinomadura*, *Mycobacterium*. In addition, the analysis found that the abundance of all 10 microorganisms with feature importance values greater than 1% generally declined with the number of continuously planted *C. equisetifolia* trees (**Figure 10A**). Therefore, in this study, *q*RT-PCR was further used to quantify microorganisms belong to *Actinoallomurus*, *Actinomadura*, *Mycobacterium* in the soil, and the results showed (**Figure 10B**) that, with the increase of the number of continuously planting *C. equisetifolia* trees, the quantity of *Actinoallomurus*, *Actinomadura*, *Mycobacterium* tended to decrease significantly, and the results verified the conclusions of the metagenomic analysis.

### 3.9 Functional analysis of characteristic microorganisms

**Figure 11:**
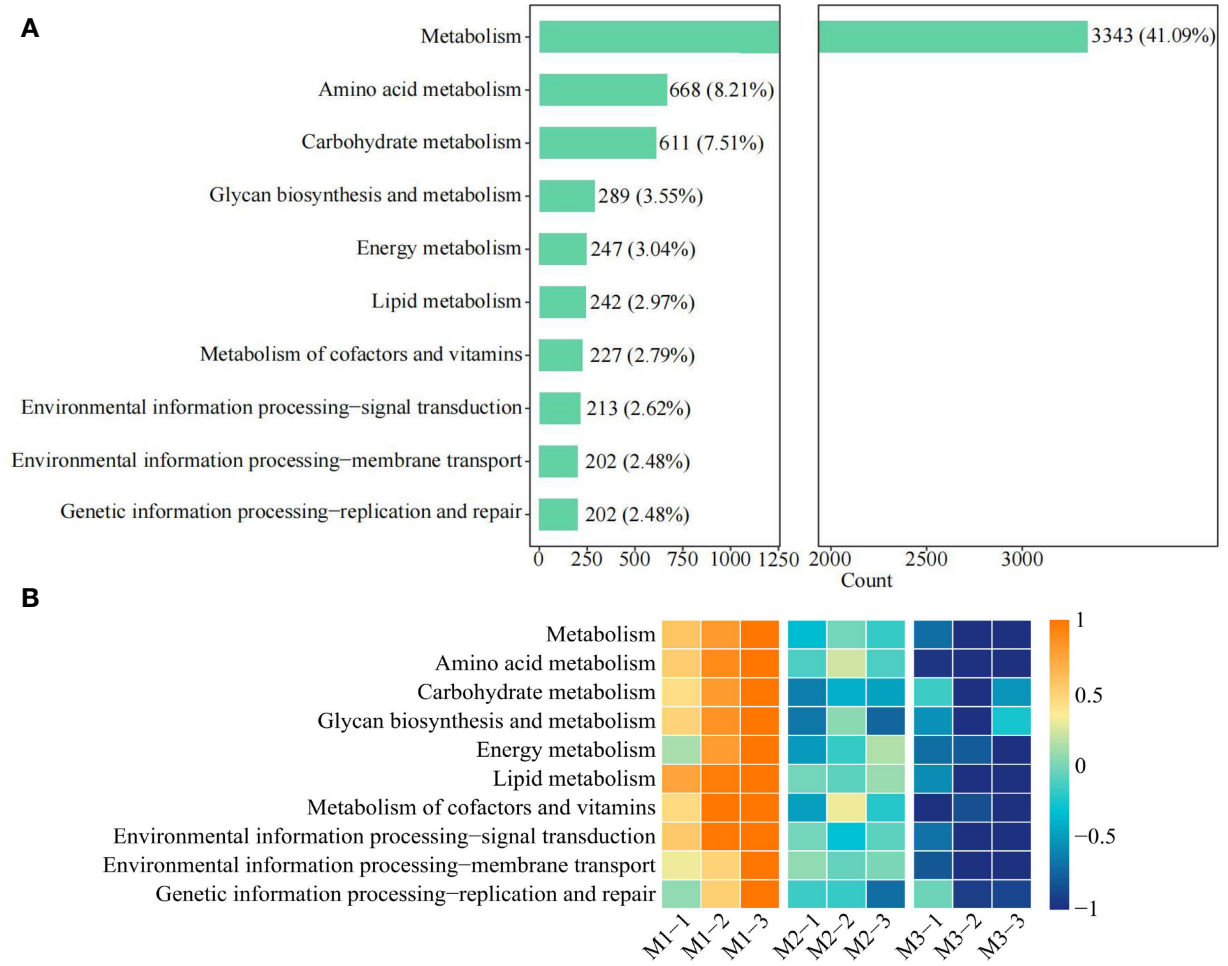
Function analysis of genes involved in characteristic microorganisms. M1: First planting; M2: Second continuous planting; M3: Third continuous planting; **(A)** Analysis of gene functions of characteristic microorganisms (only the functions of the top 10 genes are shown in the figure); **(B)** Analysis of gene expression for different functions.

Based on the previous study, this study further analyzed the functions of the 10 characteristic microorganisms, and 8,450 genes detected in the soil were from the 10 characteristic microorganisms, of which 8,135 genes were enriched to the KEGG pathway, accounting for 96.27%. Therefore, analysis of the functions of 8,135 genes revealed (**Figure 11A**) that 45 metabolic pathways were mainly involved, of which the top 10 pathways in terms of the number of genes were metabolism (41.09%), amino acid metabolism (8.21%), carbohydrate metabolism (7.51%), glycan biosynthesis and metabolism (3.55%), energy metabolism (3.04%), lipid metabolism (2.97%), metabolism of cofactors and vitamins (2.79%), environmental information processing—signal transduction (2.62%), environmental information processing—membrane transport (2.48%), genetic information processing—replication and repair (2.48%). Further analysis of the overall gene expression of the 10 metabolic pathways revealed (**Figure 11B**) that the gene expression of all 10 metabolic pathways showed a significant decreasing trend as the number of continuous planting increased. It can be seen that continuous planting led to a decrease in the metabolism and reproduction capacity of soil characteristic microorganisms, a decrease in their number, a decrease in the nutrient cycling capacity of the soil, and a decrease in the transport capacity of substances, which subsequently affected the tree growth.

### 3.10 Interacion relationship analysis

**Figure 12:**
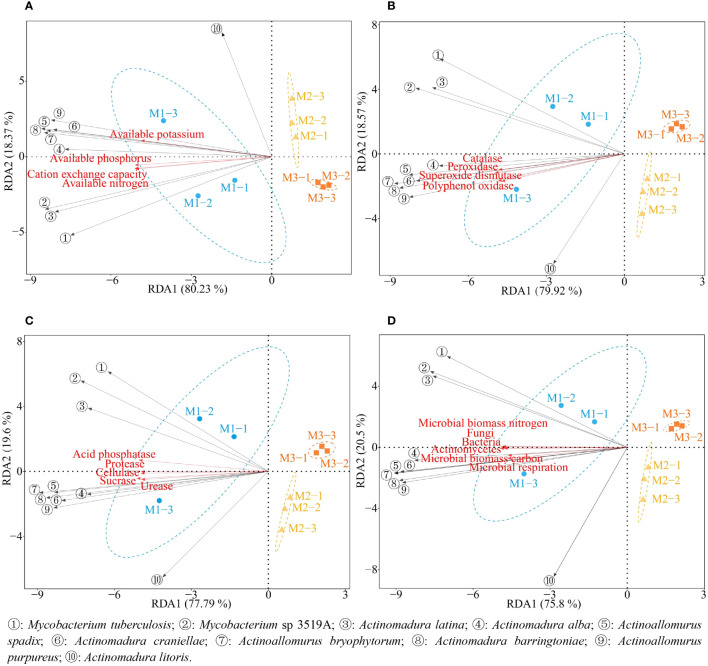
Redundancy analysis of 10 characteristic microorganisms with different indexes. M1: First planting; M2: Second continuous planting; M3: Third continuous planting. **(A)** Redundancy analysis of characteristic microorganisms and soil physicochemical property; **(B)** Redundancy analysis of characteristic microorganisms and soil resistance related enzymes; **(C)** Redundancy analysis of characteristic microorganisms and soil nutrient cycling related enzymes; **(D)** Redundancy analysis of characteristic microorganisms and soil microbial physiological indexes.

**Figure 13:**
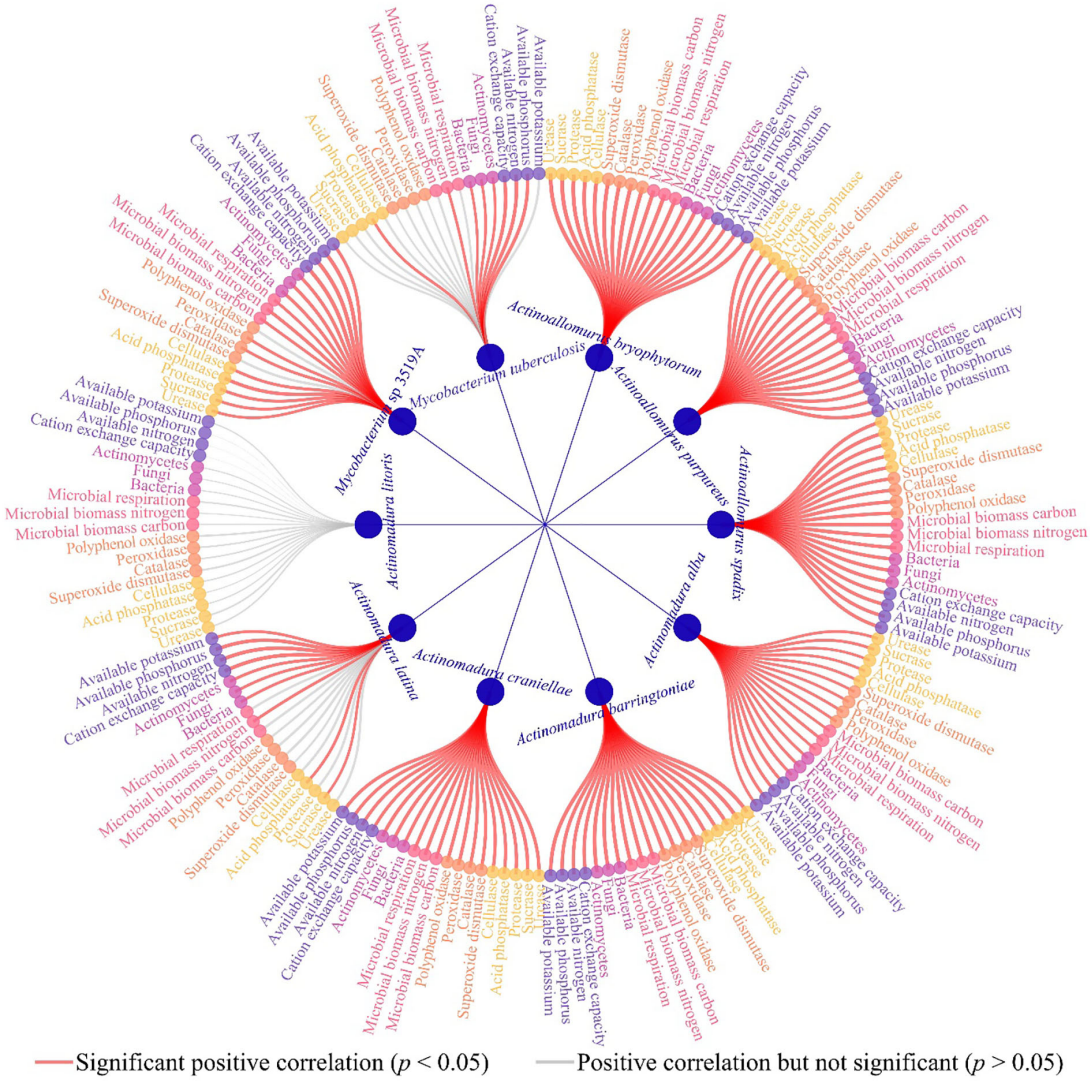
Interaction network analysis of 10 characteristic microorganisms with different indexes.

Based on previous studies, this study further analyzed the interactions between 10 characteristic microorganisms and soil physicochemical property, soil enzyme activities, and microbial physiological indexes. The results of redundancy analysis (**Figure 12**) showed that all 10 characteristic microorganisms except *Actinomadura litoris* were significantly correlated with M1, and soil physicochemical characteristics, soil resistance-related enzyme activities, soil nutrient cycling-related enzyme activities and soil microbial physicochemical indexes were all significantly correlated with M1. The results of the interactions network analysis showed (**Figure 13**) that the abundance of all 10 characteristic microorganisms was positively correlated with soil physicochemical property, soil enzyme activities and microbial physiological indexes, of which six characteristic microorganisms were significantly positively correlated with soil physicochemical property such as *Actinoallomurus bryophytorum*, *Actinoallomurus purpureus*, *Actinoallomurus spadix*, *Actinomadura alba*, *Actinomadura barringtoniae, Actinomadura craniellae* (*p* < 0.05), while the correlation of *Actinomadura litoris* did not reach the significant level (*p* > 0.05); *Actinomadura latina* with urease, protease, cellulose, superoxide dismutase, catalase, peroxidase, polyphenol oxidase, microbial biomass carbon, microbial respiration did not reach the significant level (*p* > 0.05), and reached the significant level with all other indexes (*p* < 0.05); *Mycobacterium* sp 3519A did not reach significant level of correlation with superoxide dismutase, polyphenol oxidase (*p* > 0.05), and reached significant level with all other indexes (*p* < 0.05); *Mycobacterium tuberculosis* did not reach the significant level of correlation with urease, sucrase, protease, cellulose, superoxide dismutase, catalase, peroxidase, polyphenol oxidase, microbial biomass carbon, microbial respiration, available potassium (*p* > 0.05), and reached the significant level with all other indexes (*p* < 0.05). It can be seen that the nutrient cycling capacity of the rhizosphere soil of continuously planted *C. equisetifolia* trees closely related to soil enzymes and microorganisms, especially the characteristic microorganisms.

## 4 Discussion

Long-term monoculture of plants can easily lead to deterioration of soil texture, which in turn affects plant growth (Guo et al., 2022a; Wang et al., 2022a). Soil is important for plants to obtain nutrients, and the content of nutrients in the soil and its ability to transform them directly affects plant growth, especially available nutrients (Soares et al., 2019). High available nutrient content of soil facilitates the promotion of nutrient uptake and translocation by plants, which in turn promotes plant growth, and conversely hinders plant growth (Wang et al., 2023). In this study, it was found that continuous planting did lead to severe stunting of *C. equisetifolia* seedlings growth. Secondly, CEC, AN, AP, and AK in rhizosphere soils were significantly reduced after continuous planting of *C. equisetifolia* trees. It can be seen that continuous planting leads to the weakening of the ion exchange capacity of the rhizosphere soil, the reduction of the nutrient conversion capacity, and the decrease of the available nutrient content in the soil, which in turn affects the tree growth.

Soil enzyme activity is an important index characterizing the biological status of soil and soil fertility, which indirectly reflects the effect of soil on plant growth (Zielewicz et al., 2021). Elevated activities of soil nutrient cycling-related enzymes favor the promotion of soil nutrient cycling and increase the content of available nutrients in the soil, whereas soil resistance-related enzyme activities favor the maintenance of soil health, which in turn promotes nutrient uptake by plant roots (Zhang et al., 2023a). In this study, it was found that the activity of rhizosphere soil nutrient cycling enzymes (urease, sucrase, protease, acid phosphatase, and cellulase) and resistance-related enzymes (superoxide dismutase, catalase, peroxidase, and polyphenol oxidase) showed a significant decreasing trend as the number of continuous plantings of *C. equisetifolia* trees increased. It has been reported that soil urease and protease activities are related to nitrogen cycling in the soil, and increasing their activity contributes to the increase of available nitrogen content in the soil (Jia et al., 2023a). Enhanced acid phosphatase activity is beneficial to increase available phosphorus content in soil (Tian et al., 2021), while enhanced sucrase and cellulase activity is beneficial to increase soil carbon cycle, promote microbial growth, and improve soil nutrient cycling capacity (Zheng et al., 2023). Superoxide dismutase, catalase, peroxidase and polyphenol oxidase have been reported to be closely related to soil health, and increasing their activity is beneficial to maintaining soil health and promoting microbial propagation and plant root growth in soil (Lin et al., 2022). It can be seen that continuous planting led to a decrease in the activities of rhizosphere soil nutrient cycling-related enzymes and resistance-related enzymes, which in turn reduced the available nutrient content of the soil, fission of the soil texture, and impeded the tree growth.

Plant-microbe-soil is an interacting organism, where microorganisms can change the environment of the soil, which in turn affects the growth of plants, and at the same time, environmental changes can affect microbial propagation, which in turn affects microbial quantity and community diversity (Mącik et al., 2020; Zhong et al., 2020). In this study, it was found that the total number of soil microorganisms in the rhizosphere zone showed a decreasing trend with the increase in the number of continuous plantings of *C. equisetifolia* trees, but the number of fungi showed an increasing trend. It has been reported that continuous planting of plants is highly susceptible to shifting soil from bacterial to fungal type, which in turn leads to a decline in soil microbial quantity, growth of pathogenic bacteria, and hidered plant growth (Liu et al., 2019). It can be seen that continuous planting reduced the cycling capacity of microbial biomass carbon and nitrogen in rhizosphere soil, decreased the quantity of soil bacteria and actinomycetes, but increased the quantity of soil fungi, and the structure of the microbial community was significantly changed. Accordingly, this study further analyzed the effect of continuous planting on the structure and function of soil microbial community in the rhizosphere soil of *C. equisetifolia* trees by using metagenomics technology, and found that the α-diversity indexes and β-diversity of soil microbial community in the rhizosphere soil of *C. equisetifolia* trees were significantly changed after continuous planting. The α-diversity indexes are important in the assessment of microbial community diversity, where the richness and chao1 indexes can be used to assess species richness, with larger indexes indicating greater species richness, and simpson and shannon are used to assess the diversity of species within a sample (Willis, 2019). β-diversity can be used to assess differences in species diversity between samples (Xiao et al., 2021). It can be seen that continuous planting has altered the microbial community structure and abundance in the rhizosphere soil of *C. equisetifolia* trees. Therefore, in this study, OPLS-DA combined with XGBoost technique was further applied to screen the characteristic microorganisms that caused significant changes in the rhizosphere soil of *C. equisetifolia* trees through continuous planting. The results showed that 10 characteristic microorganisms from *Actinoallomurus*, *Actinomadura* and *Mycobacterium* were found, and their abundance in general showed a significant decreasing trend with the number of continuously planted *C. equisetifolia*. Microorganisms of *Actinoallomurus* have been reported to be associated with nitrogen cycling in soil, and continuous planting reduces the microbial abundance of *Actinoallomurus* sp. in soil, which in turn reduces the activity of nitrogen cycling-related enzymes, and reduces the content of available nutrients (Tang et al., 2020; Zheng et al., 2022). Secondly, *Actinoallomurus* sp. belong to actinomycetes, which have the ability to secrete antimicrobial metabolites that can effectively inhibit pathogens in the soil, prevent plants from being infected by pathogens, and promote plant growth (Mast and Stegmann, 2019; Iorio et al., 2022). *Actinomadura* is conducive to improving cycling capacity of soil C and N and increasing soil available nutrient content, however, continuous planting reduces *Actinomadura* abundance, which in turn hinders soil nutrient cycling and inhibits plant growth (Zhang et al., 2020; Lu et al., 2023). Meanwhile, *Actinomadura* has a strong antimicrobial sensitivity and can effectively inhibit soil pathogens and promote plant growth (Li et al., 2022; Watson et al., 2022). *Mycobacterium* has an important role in degrading soil hazardous substances, and a large number of studies have shown that increasing the abundance of *Mycobacterium* sp in soil can reduce the content of different types of soil hazardous substances, improve soil texture, and promote soil nutrients cycling for healthy plant growth (Kim et al., 2013; Kandil et al., 2015; Li et al., 2021a; Zhao X. et al., 2021). It can be seen that continuous planting led to a decrease in the abundance of *Actinoallomurus* and *Actinomadura* in the rhizosphere soil, a decrease in the nutrient cycling capacity of the rhizosphere soil, a decrease in the content of available nutrients, and an impact on the *C. equisetifolia* tree growth; second, continuous planting led to a decrease in the abundance of *Mycobacterium* in the *C. equisetifolia* tree rhizosphere soil, which reduced the degradation capacity of harmful substances, increased the accumulation of harmful substances in the soil, and hindered the *C. equisetifolia* tree growth.

Further analysis of the functions of 10 characteristic microorganisms revealed that with the increasing number of continuous planting, the environmental information processing-signal transduction capacity of soil characteristic microorganisms was weakened, and the ability of *C. equisetifolia* trees to defend itself against stress caused by continuous planting decreased. Secondly, reduced capacity of metabolism, genetic information processing-replication and repair led to a decrease in microbial propagation in rhizosphere soil and a reduction in the number of microorganisms in *C. equisetifolia* trees. However, amino acid metabolism, carbohydrate metabolism, glycan biosynthesis and metabolism, lipid metabolism, metabolism of cofactors and vitamins were significantly reduced in the characteristic microorganisms of *C. equisetifolia* tree rhizosphere soil after continuous planting were significantly reduced, indicating that continuous planting tended to reduce the synthesis and metabolism of carbon, nitrogen, lipids, vitamins, and sugars of the characteristic microorganisms in rhizosphere soil, as well as the nutrient cycling capacity of the soil. In addition, it was found that environmental information processing-membrane transport of soil was significantly reduced after continuous planting, indicating that continuous planting resulted in a decrease in the capacity of rhizosphere soil to transport substance. It can be seen that the number of 10 characteristic microorganisms decreased significantly after continuous planting, which led to a decrease in the activities of soil resistance-related enzymes and nutrient cycling-related enzymes, a decrease in the nutrient conversion capacity of the soil, and a decrease in the content of available nutrients in the soil, which in turn impeded the growth of *C. equisetifolia* trees, especially the six characteristic microorganisms of *Actinoallomurus* and *Actinomadura*, including *Actinoallomurus bryophytorum*, *Actinoallomurus purpureus*, *Actinoallomurus spadix*, *Actinomadura alba*, *Actinomadura barringtoniae*, *Actinomadura craniellae* (**Figure S2**).

## 5 Conclusion

**Figure 14:**
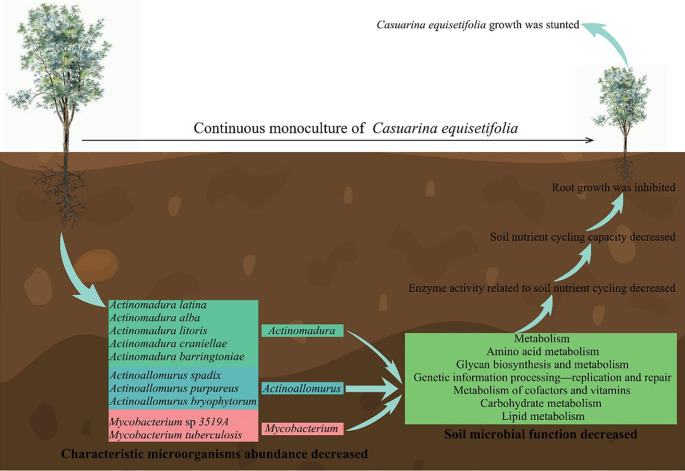
Mechanism analysis of effects of continuous planting on rhizosphere soil biology of *Casuarina equisetifolia*.

In this study, it was found (**Figure 14**) that continuous planting resulted in dwarfing, shorter root length and reduced root system of *C. equisetifolia* seedlings, significantly affecting the growth of *C. equisetifolia* trees and increasing with the number of continuous plantings. Second, continuous planting led to a significant decrease in the abundance of 10 characteristic microorganisms of *Actinoallomurus*, *Actinomadura* and *Mycobacterium* in rhizosphere soil, which in turn reduced gene expression in metabolism, environmental information processing-signal transduction, genetic information processing-replication and repair, which reduced the ability of microorganisms to defend against environmental impacts and led to a decrease in soil resistance-related enzymes and microbial quantity. Third, continuous planting decreased the gene expression of 10 characteristic microorganisms, namely amino acid metabolism, carbohydrate metabolism, glycan biosynthesis and metabolism, lipid metabolism, metabolism of cofactors and vitamins, and reduced the synthesis and metabolism of soil carbon, nitrogen, lipids, vitamins and sugars, which in turn reduced the activity of soil nutrient cycling-related enzymes, and the available nutrient content of the soil in the *C. equisetifolia* rhizosphere declined, the ion-exchange capacity was weakened, and the growth was impeded. In this study, we analyzed the causes of growth retardation due to continuous planting from the perspective of rhizosphere soil microorganisms, which provided an important reference for *C. equisetifolia* cultivation management. In the management process of continuous planting of *C. equisetifolia* plants, it is necessary to pay attention to soil nutrient conversion capacity, reasonable fertilization to improve soil nutrient conversion, in order to ensure that *C. equisetifolia* has effective access to nutrients, and then to protect growth. Meanwhile, 10 characteristic microorganisms were obtained in this study, which will be screened and colonized to further explore the effects of characteristic microorganisms on *C. equisetifolia* growth with continuous planting. The results of the study laid the foundation for subsequent bacterial fertilizer to regulate the growth of continuously planted *C. equisetifolia*.

## Data availability statement

The original contributions presented in the study are included in the article/**Supplementary Files**, further inquiries can be directed to the corresponding author/s.

## Author contributions

YW: Conceptualization, Formal analysis, Methodology, Visualization, Writing – original draft, Writing – review & editing. SL: Conceptualization, Formal analysis, Methodology, Visualization, Writing – original draft, Writing – review & editing. JL: Investigation, Methodology, Writing – original draft. XJ: Formal analysis, Writing – review & editing. MH: Investigation, Methodology, Writing – original draft. YCa: Investigation, Methodology, Writing – original draft. PC: Investigation, Methodology, Writing – original draft. ML: Formal analysis, Writing – review & editing. YCh: Formal analysis, Writing – review & editing. WL: Formal analysis, Writing – review & editing. HW: Conceptualization, Formal analysis, Funding acquisition, Methodology, Project administration, Resources, Supervision, Visualization, Writing – original draft, Writing – review & editing. ZW: Conceptualization, Formal analysis, Funding acquisition, Methodology, Project administration, Resources, Supervision, Visualization, Writing – original draft, Writing – review & editing.
