# Supplementary material for: Metagenomics-based exploration of key soil microorganisms contributing to continuously planted Casuarina equisetifolia growth inhibition and their interactions with soil nutrient transformation
Source: Front Plant Sci. 2023 Dec 6;14:1324184. doi: 10.3389/fpls.2023.1324184 (PMC10731376; doi:10.3389/fpls.2023.1324184)
Supplement: Supplementary file 1 [file DataSheet_1.pdf]

Supplementary data

Table S1 Statistical table of basic information of sequencing data

Sample	CleanData bases (G)	CleanData Q20	CleanData Q30	CleanData GC
M1-1	6.5813	98.11	94.4	64.91
M1-2	6.9357	98.035	94.23	65.47
M1-3	7.3408	98.06	94.265	65.04
M2-1	6.1252	97.47	92.88	63.83
M2-2	9.1357	98.065	94.265	64.26
M2-3	7.6467	98.02	94.165	63.945
M3-1	7.4075	97.755	93.465	64.415
M3-2	7.1387	97.86	93.73	63.53
M3-3	6.5434	97.725	93.445	63.96

Note: M1: First planting; M2: Second continuous planting; M3: Third continuous planting; Sample: Sample name; CleanData: Indicates valid data obtained by filtering; Bases: Number of bases in the data; Q20: Represents the percentage of the number of bases in the data with a sequencing error rate of less than 0.01(mass value greater than 20); Q30: Represents the percentage of the number of bases in the data with a sequencing error rate less than 0.001(mass value greater than 30); GC: GC content of bases in the data.

Table S2 Statistical table of basic information of contigs of assembly results of each sample

Assembly	Sample								
	**M1-1**	**M1-2**	**M1-3**	**M2-1**	**M2-2**	**M2-3**	**M3-1**	**M3-2**	**M3-3**
Contigs (≥ 0 bp)	274650	284635	284035	177209	297075	246549	219578	199365	195339
Contigs (≥ 500 bp)	274650	284635	284035	177209	297075	246549	219578	199365	195339
Contigs (≥ 1000 bp)	54500	54968	55349	26016	48114	37099	41415	39130	38171
Contigs (≥ 5000 bp)	1828	1868	2008	720	946	816	860	846	581
Contigs (≥ 10000 bp)	348	301	456	182	340	278	206	153	115
Contigs (≥ 25000 bp)	16	8	25	3	59	26	58	33	50
Contigs (≥ 50000 bp)	1	1	1	0	2	0	30	23	37
Length (≥ 0 bp)	245282620	251176495	253849982	142158393	244320775	198454284	192837065	178293440	172148305
Length (≥ 500 bp)	245282620	251176495	253849982	142158393	244320775	198454284	192837065	178293440	172148305
Length (≥ 1000 bp)	100502620	99958550	103541962	43836521	81357562	61737737	75189208	72570634	68506596
Length (≥ 5000 bp)	14857216	14517360	17213161	6189120	10277592	8061963	11945261	11510597	9026513
Length (≥ 10000 bp)	4933663	4023874	6774587	2492581	6205535	4357821	7678633	6983439	5965165
Length (≥ 25000 bp)	549223	255293	762518	96423	1962414	777344	5515694	5318409	5058357
Length (≥ 50000 bp)	53058	53097	56290	0	104015	0	4544011	4981917	4636710
Total contigs	274650	284635	284035	177209	297075	246549	219578	199365	195339
Largest contig	53058	53097	56290	35086	52410	37694	697671	750474	379347
Total length	245282620	251176495	253849982	142158393	244320775	198454284	192837065	178293440	172148305
GC (%)	65.69	66.71	66.13	65.21	65.43	65.22	65.68	65.12	65.13
N50	851	838	848	751	776	756	831	848	839
N75	622	620	622	593	602	597	623	627	624
L50	78604	83108	80852	57973	94987	80856	64859	57215	57452
L75	164249	171461	169711	111778	185355	155493	132836	119381	117932

Note：M1: First planting; M2: Second continuous planting; M3: Third continuous planting; Sample: Sample name; Contigs: Indicates the number of contigs obtained by assembly; Length: Represents the length of the assembled contigs; Total contigs: The total number of contigs assembled; Largest contig: Indicates the maximum length of contigs; Total length: Indicates the total length of contigs assembled; N50(N75): Indicates that contigs are sorted by length, and then the sum is added from length to length, when the sum value reaches 50%(75%) of the total length of contigs; L50(L75): Indicates the number of contig when it reaches N50(N75).

Table S3 Statistical table of basic information of Unigenes

List	Unigenes
ORFs number	2202069
Integrity-start	675721 (30.69%)
Integrity-end	672437 (30.54%)
Integrity-none	285975 (12.99%)
Integrity-all	567936 (25.79%)
Total Length (Mbp)	1010.4
Average Length (bp)	458.84
GC percent	65.89

Note： ORFs number: Indicates the number of genes in sample; Integrity-start: Represents the number and percentage of genes that contain only the start codon; Integrity-end: Represents the number and percentage of genes that contain only stop codons; Integrity-none: Represents the number and percentage of genes with neither start nor stop codons; Integrity-all: Represents the number and percentage of complete genes (both start and stop codons); Total Length: The total length of a gene catalogue; Average Length: Indicates the average length of genes in the gene catalogue; GC Percent: Represents the total GC content of the gene in the predicted gene catalogue.

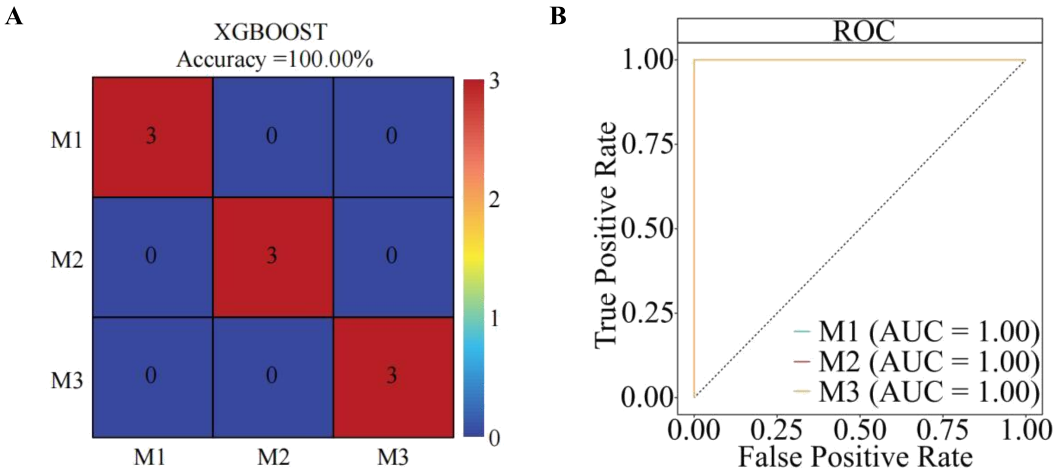

Fig. S1 XGBoost machine learning simulation curve analysis

M1: First planting; M2: Second continuous planting; M3: Third continuous planting; A: Confusion matrix diagram for XGBoost machine learning simulation validation; B: ROC curve for XGBoost machine learning simulation validation.

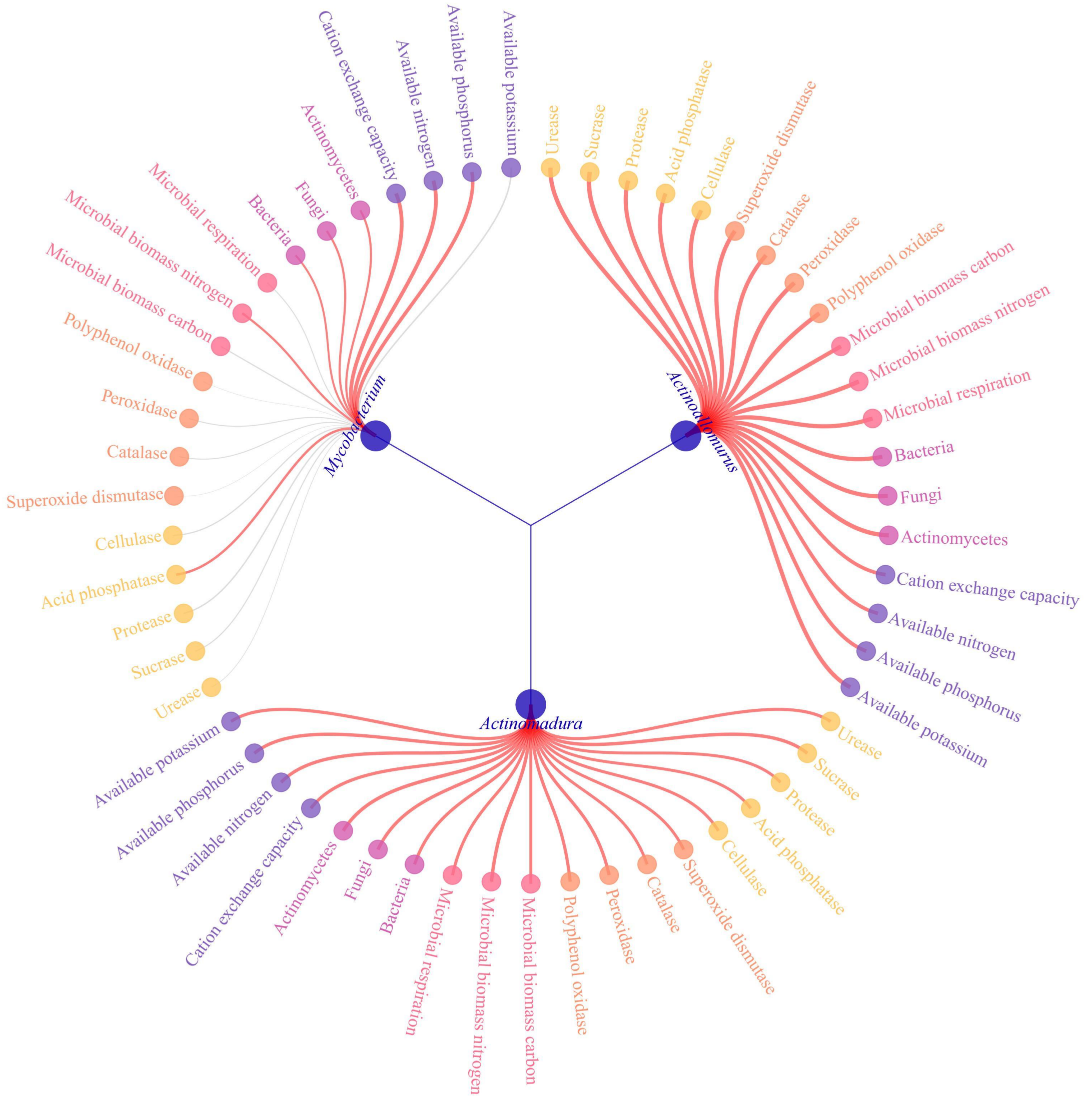

Significant positive correlation ($p < 0.05$) Positive correlation but not significant ($p > 0.05$)

Fig. S2 Interaction network analysis of *Actinoallomurus*, *Actinomadura*, and *Mycobacterium* with different indexes
